# Long-Term Safety and Real-World Effectiveness of Trastuzumab in Breast Cancer

**DOI:** 10.3390/jcm8020254

**Published:** 2019-02-18

**Authors:** Marco Mazzotta, Eriseld Krasniqi, Giacomo Barchiesi, Laura Pizzuti, Federica Tomao, Maddalena Barba, Patrizia Vici

**Affiliations:** 1 Department of Clinical and Molecular Medicine, “Sapienza” University of Rome, Azienda Ospedaliera Sant’Andrea, 00189 Rome, Italy; mmazzotta@ospedalesantandrea.it; 2 Division of Medical Oncology 2, IRCCS Regina Elena National Cancer Institute, Via Elio Chianesi, 53, 00144 Rome, Italy; krasniqier@gmail.com (E.K.); giacomo.barchiesi88@gmail.com (G.B.); laura.pizzuti@ifo.gov.it (L.P.); patrizia.vici@ifo.gov.it (P.V.); 3 Department of Gynecology-Obstetrics and Urology, “Sapienza” University of Rome, 00161 Rome, Italy; federica.tomao@uniroma1.it

**Keywords:** HER2 positive breast cancer, trastuzumab, long-term safety, efficacy, real-world settings

## Abstract

Trastuzumab is a milestone in the treatment of human epidermal growth factor receptor 2 positive (HER2+) breast cancer (BC), in both the early and metastatic settings. Over the last two decades, clinical trials have established the good safety profile of trastuzumab. Cardiotoxicity remains the most frequent adverse event, more commonly exemplified by an asymptomatic decline in the left ventricular ejection fraction rather than congestive heart failure. Results from several long-term (>5 years) safety analyses have been recently published, with the inherent evidence substantially confirming the findings from previous trials. The clinical experience gained over the years in the use of trastuzumab has also fueled a number of observational studies focused on the effectiveness of this drug in the real-world settings. We herein reviewed the evidence available from tree major databases, namely, PubMed, EMBASE and the Cochrane Central Register of Controlled Trials (CENTRAL), to explore and critically discuss key issues related to the long-term safety and effectiveness of trastuzumab in clinical practice.

## 1. Introduction

Trastuzumab is a humanized monoclonal antibody directed against the extracellular domain of human epidermal growth factor receptor 2 (HER2)-neu receptor. It represents the standard of care in breast cancer (BC) patients with HER2 amplification and/or overexpression both in the advanced and (neo)adjuvant settings. The amplification/overexpression of HER2 receptor can be identified in approximately 20% of invasive breast neoplasms [1]. HER2-positive (HER2+) BC is characterized by a biologically aggressive behavior and worse prognosis when compared to HER2 negative tumors. Prior to the availability of anti-HER2 therapies, women diagnosed with early stage HER2+ BC experienced a significantly shorter time to relapse, higher mortality and increased incidence of metastases [2]. Adding trastuzumab to standard chemotherapy has led to a significant improvement in survival outcomes [3]. Currently, patients with HER2+ BC are treated worldwide with a combination of chemotherapy plus trastuzumab and/or other target therapies against HER2. Various HER2 targeted agents (pertuzumab, trastuzumab-emtansine, lapatinib and neratinib) have been developed in recent years and are now used in the metastatic or (neo)adjuvant settings, in combination with or following trastuzumab treatment. However, nowadays trastuzumab remains widely used as a single agent all over the world. Moreover, several trastuzumab biosimilar drugs have also been developed and are currently available in clinical practice [4,5]. Despite the overall encouraging safety profile of trastuzumab, a peculiar adverse event has emerged and caught the attention of the scientific and patient community: cardiotoxicity. There is an ample amount of evidence on trastuzumab toxicity, which is generally consistent across all the international trials. However, data from long-term follow up studies have been collected and examined only in the few latest years. Furthermore, several observational studies are now available reporting data of patients with HER2+ disease with a focus on specific features, e.g., hormone receptors’ expression and specific patients’ subsets. The review herein proposed aims at exploring aspects related to trastuzumab long-term safety profile and actual effectiveness of trastuzumab treatment in HER2+ BCs from the real-world setting.

## 2. Materials and Methods 

A literature review was conducted based on the guidance of the Centre for Reviews and Dissemination (Systematic reviews: CRD’s guidance for undertaking reviews in healthcare. York: Centre for Reviews and Dissemination, 2009). An iterative approach was used starting with an electronic search of the MEDLINE, EMBASE and the Cochrane Central Register of Controlled Trials (CENTRAL) databases (DBs) (customized range date until 30 November 2018). Both text words and Medical Subject Headings (MeSH) thesaurus were adopted to perform a more tailored search strategy. Since this review was designed to explore two distinct aspects, i.e., long-term safety profile and real-world effectiveness of trastuzumab, we proceeded in a dichotomic search. Literature assessment was performed by 4 of the authors (MM, GB, LP, and MB).

### 2.1. Long-Term Safety

First, the search terms “((trastuzumab [MeSH Terms]) AND breast cancer [MeSH Terms]) AND long-term safety” were used. Citation tracking and search were performed in the previously mentioned DBs.

We considered for inclusion phase II and III randomized controlled trials (RCTs), and observational studies (OSs) published within the last 10 years. Intervention trials had to include a trastuzumab treatment arm and OSs had to be related to patients having received trastuzumab. Independently on the study design, the required follow up period following trastuzumab administration was of at least five years. We also evaluated systematic reviews and meta-analyses of phase II-III RCTs and/or OSs with the aforementioned characteristics. Exclusion criteria were as follows: tumors other than BC; treatment with HER2 target agents other than trastuzumab or combining trastuzumab with other HER2 targeted agents; we also excluded case reports, case series, reviews, guidelines or consensus articles, and letters to the editor. The full-text version of the manuscripts selected were obtained and the related references screened manually for additional relevant articles.

**Figure 1:**
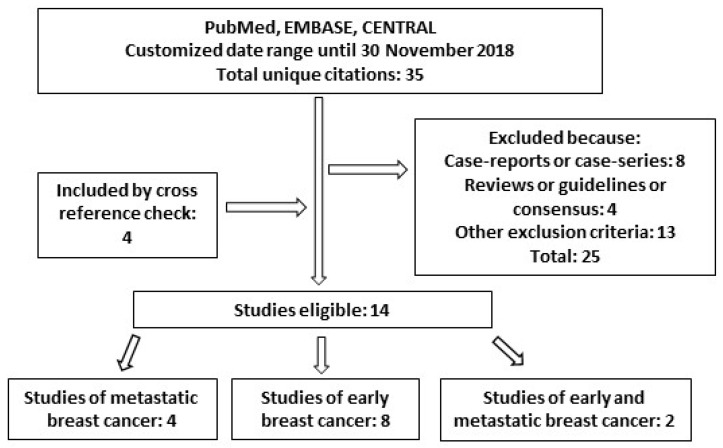
Diagram of studies found for “long-term safety” of trastuzumab in this review.

Overall, the search performed in PubMed, EMBASE and CENTRAL yielded a total of 35 unique citations. Additional publications suitable for inclusion were found by cross-reference check. Based on the aforementioned inclusion and exclusion criteria, 14 papers resulted eligible for analysis (Figure 1, Table 1). Among them, 4 studies were focused on metastatic breast cancer, 8 were inherent to the (neo)adjuvant setting and 2 included data on both.

### 2.2. Real-World Effectiveness

The search terms “(trastuzumab [MeSH Terms]) AND breast cancer [MeSH Terms]) AND/OR real-world” were used. Citation tracking and search were performed in the aforementioned DBs. 

Inclusion criteria were as follows: observational studies with a prospective, retrospective or mixed design focused on patients from the real-word setting and published within the last 10 years; treatment with trastuzumab was required. Exclusion criteria were: treatment with HER2 targeted agents other than trastuzumab, singularly or in combination with trastuzumab; articles concerning cost-effectiveness or other economic/financial studies, case reports, guidelines or consensus articles, and letters to the editor. The full-text versions of the search results were obtained and the inherent reference lists were screened for additional relevant manuscripts.

**Figure 2:**
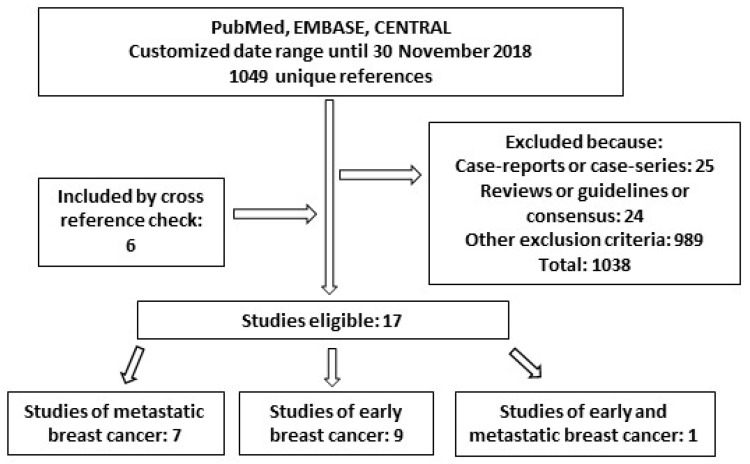
Diagram of studies found for “real-world effectiveness” of trastuzumab in this review.

Adding the filter “observational study” to the search, a total of 1049 unique references were identified based on the search performed in PubMed, CENTRAL and EMBASE DBs. Other publications concerning our research question were found by cross-reference check. Overall, 17 papers resulted eligible for analysis (Figure 2, Table 2). Among them, seven studies were about metastatic breast cancer, while nine were inherent to the (neo)adjuvant setting and one to both.

## 3. Long-Term Safety

Data from intervention trials showed an excellent safety profile for trastuzumab, except for cardiotoxicity. This latter is most commonly associated with an asymptomatic decline in the left ventricular ejection fraction (LVEF) rather than a congestive heart failure (CHF) [33]. On this basis, all the studies focusing on the safety profile of patients treated with trastuzumab also investigated the cardiac toxicity. We herein report the studies included in this paper (Table 1).

Despite considerable efforts, the mechanisms responsible for trastuzumab-related cardiotoxicity have not been fully elucidated yet. The most likely explanation is that trastuzumab interferes with the action of Neuregulin (NRG), a protein expressed in the microvascular endothelium with both antioxidant and antiapoptotic properties. At the myocyte level, it also interacts with myocardial metabolism through the activation of ErbB receptors, which include HER2. The inhibition of ErbB2 operated by trastuzumab may reduce the protective effects of NRG on myocardium [34]. Secondly, it has been supposed that the HER2 blockage by trastuzumab leads to the accumulation of reactive oxygen species (ROS), which cause cell damage and prelude to cell apoptosis [35,36].

### 3.1. Early Breast Cancer

Data on long-term safety concerning the use of trastuzumab in early breast cancer (eBC) are available from the most recent updates of the pivotal trials conducted in this setting.

The HERA trial (BIG 1-01) was the first trial to investigate the role of trastuzumab in the adjuvant setting. It was an international, multicentre, open-label, phase 3 randomized trial that enrolled 5102 women with HER2+ eBC, between 2001 and 2005 [37]. Following surgery, adjuvant chemotherapy and radiotherapy, patients were randomly assigned (1:1:1) to receive trastuzumab for 1 year or 2 years, or to the observation group. The study demonstrated that a 1-year treatment with trastuzumab significantly improved disease free survival (DFS) compared to the observation group. No survival difference emerged between the 1-year and 2-year treatment groups. Regarding the safety profile, cardiac toxicity was more frequent in the trastuzumab groups compared to the observation one. Cardiac adverse events leading to discontinuation of trastuzumab occurred in 9.4% of patients in the 2-year arm and 5.2% of patients in the 1-year arm. Primary cardiac events were symptomatic congestive heart failure, defined as New York Heart Association (NYHA) class III or IV, a clinically significant LVEF drop of at least 10 percentage points from baseline, an absolute LVEF below 50%, or death from cardiac causes. Such events were on average poorly represented in all groups (two [0.1%] in the observation group, 18 [1%] in the 1-year trastuzumab group and 17 [1%] in the 2-year trastuzumab group). Secondary cardiac endpoints were defined as asymptomatic [NYHA class I] or mildly symptomatic [NYHA class II] with a clinically significant LVEF drop of at least 10 percentage points from baseline and an absolute LVEF below 50% confirmed by repeated assessment. These latter events occurred more frequently in the 2-year trastuzumab group (122 [7·3%]) than in the 1-year trastuzumab group (74 [4.4%]) and in the observation group (15 [0.9%]). Cardiac events occurred predominantly during trastuzumab treatment, as demonstrated in the analysis published in 2014 at 8 years of median follow-up, with the majority of patients achieving rapid recovery [38]. The 11-year follow up analysis, published in 2017, showed no evidence of significant change in the number of cardiac events occurring after a longer time since randomization [6].

Another key trial of trastuzumab in eBC was the National Surgical Adjuvant Breast and Bowel Project (NSABP) Protocol B-31, wherein the 1830 patients enrolled received anthracycline and taxane chemotherapy with or without trastuzumab for adjuvant treatment of node-positive, HER2+ eBC [7]. After a seven-year follow up, data on the cardiac assessment of the patients included was published. Thirty-seven out of 944 subjects in the trastuzumab arm experienced a cardiac event versus 10 out of 743 patients in the control one [39]. As described above, the majority of patients recovered LVEF in the normal range, although some decline from baseline persisted. Two cardiac deaths were registered, one per cohort. Only two cardiac events were registered after 2 years of trastuzumab treatment. In 2017, at a median follow-up of 8.8 years, Ganz and colleagues published a long-term safety update in patients that experienced a cardiac event during the NSABP Protocol B-31: five patients (4.5%) in the control group and 10 (3.4%) in the trastuzumab group had a permanent ≥10% LVEF decline compared to baseline and a resulting absolute LVEF value <50%. According to the authors, the addition of trastuzumab did not result in long-term worsening of cardiac function [40].

The North Central Cancer Treatment Group (NCCTG) N9831 trial was an international study that randomly assigned eBC patients with operable, HER2+ disease to receive four cycles of doxorubicin and cyclophosphamide (AC) followed by either paclitaxel alone (arm A), or paclitaxel with trastuzumab either sequentially (arm B) or concurrently (arm C) [7]. The 6-year cumulative incidence of cardiac events was 0.6% in arm A, 2.8% in arm B, and 3.4% in arm C. An update of the cardiac safety analysis of the N9831 trial after a median follow-up time of 9.2 years was published in 2016: only two additional cardiac events were registered. The authors defined that the late development of cardiac events was infrequent [41].

The BCIRG 006 study randomly assigned 3222 women with HER2+ eBC to receive AC followed by docetaxel (T) every 3 weeks (AC-T), the same regimen plus 52 weeks of trastuzumab (AC-T plus trastuzumab), or docetaxel and carboplatin plus 52 weeks of trastuzumab (TCH). At a median follow up of 65 months, the rates of CHF and cardiac dysfunction were significantly higher in the group receiving AC-T plus trastuzumab (2.0%) than in the TCH (0.4%) group (*p* < 0.001). It is noteworthy that eight cases of acute leukemia were diagnosed in the anthracycline-based treatment group. In this study, the subclinical toxic effects persisted for several years: 33% of patients experienced a LVEF reduction for at least 4 years after the end of therapy [8].

In the FinHer trial, trastuzumab was added in HER2+, eBC patients after several combinations of adjuvant chemotherapy. Differently from other studies, the duration of trastuzumab treatment was 9 weeks only. At a 5-year follow up, the median LVEF of patients treated with trastuzumab did not change and, unexpectedly, the incidence of cardiac events was higher in the non-trastuzumab group than in the trastuzumab one [42].

Evidence regarding the safety of trastuzumab can also be derived from several observational studies which specifically addressed long-term toxicity in HER2+ eBC. Recently, the final analysis of cardiac events in the OHERA study, who included 3733 patients with eBC treated with trastuzumab, has been published [10]. Symptomatic CHF occurred in 106 patients (2.8%) and median time onset was 5.7 months (95% CI 5.3–6.5). As expected, 77/106 (72.6%) patients with symptomatic CHF achieved resolution. The CHF incidence was higher in patients ≥65 years, those with pre-existing cardiac conditions, hypertension, or LVEF ≤55% at baseline.

Goldhar and colleagues reported on the temporal risk of heart failure associated with adjuvant trastuzumab in 3371 breast cancer patients. After a median follow up of 5.9 years, patients treated with trastuzumab and chemotherapy were more likely to develop heart failure (HF) than patients treated with chemotherapy alone (5-year cumulative incidence of 5.2% vs. 2.5%; log-rank *p* < 0.001). Trastuzumab remained independently associated with higher incidence of HF in the first 1.5 years, but not thereafter. The authors concluded that a routine intensive monitoring may not be necessary after completing adjuvant therapy [11].

A Dutch experience provided data from 230 patients treated with trastuzumab in the adjuvant setting. With a median follow up of 5 years, asymptomatic decrease in LVEF was observed in nine patients (3.9%), severe cardiotoxicity was registered in 20 patients (8.7%) and 12 patients had a permanent drop in LVEF during the first 6 months after start of trastuzumab treatment [12].

### 3.2. Metastatic Breast Cancer

Data on long-term safety in metastatic breast cancer (MBC) setting are fewer than eBC. This is intuitively due to the shorter follow up period reached in this setting.

The LHORA study was a French trial which described the clinical features of long responder (PFS > 3 years) patients to a first-line trastuzumab therapy for HER2+ MBC. No trastuzumab-related deaths were observed. In the safety analysis (*n*= 134 patients), only three cardiac adverse events considered related to trastuzumab were recorded (2.2%) [13].

Rossi and colleagues reported on the results of an Italian observational study including 681 MBC patients treated with trastuzumab. The incidence of cardiac events increased year by year, reaching a value of 2.4% and 4.3% during the first and second year. Age was a strong predictor of cardiotoxicity. The incidence of cardiotoxicity in this cohort was higher than that reported in clinical trials [14].

The Rollover Protocol (ROP) Study was conducted to investigate safety in patients with HER2+ locally advanced/metastatic breast cancer who had received long-term trastuzumab therapy (≥5 years) in previous clinical trials. Only 19 MBC were included. No serious cardiac adverse events or relevant changes in LVEF, neither serious adverse event related to the study treatment were registered [15].

Another retrospective trial included 215 patients with HER2+ MBC who started first-line trastuzumab-containing therapy for metastatic disease between 2001 and 2010 at the Royal Marsden Hospital. Twenty-eight (13%) patients recorded any grade of left ventricular dysfunction. There was no significant difference in cardiac toxicity between patients receiving trastuzumab for less than one year vs. more than one year [16].

### 3.3. Early and Metastatic Breast Cancer

Two studies included in this review reported data on both MBC and eBC patients. Huang Ping and colleagues examined the long-term clinical tolerance and cardiac safety of trastuzumab treatment in 94 BC patients. The duration of trastuzumab treatments ranged from 3 to 60 months. Declines in LVEF ≥15% were seen mainly after 3–15 months of trastuzumab treatment [17].

Another retrospective study was conducted to assess long-term clinical tolerance and cardiac safety during trastuzumab treatment. One hundred and five patients were included. A decline of LVEF was correlated with the cumulative dose of anthracyclines administered, the use of cardiovascular drugs and duration of trastuzumab treatment [18].

## 4. Real World Effectiveness

The second objective of this review is to explore the real-world effectiveness of trastuzumab treatment in breast cancer. The literature included in following sections has been presented and critically discussed across three main domains represented by: (1) Studies of HER2+ eBC, including patients having received neoadjuvant and/or adjuvant therapy, (2) studies of HER2+ MBC, and (3) studies including both early and metastatic breast cancer patients (Table 2).

### 4.1. Early Breast Cancer

A large retrospective study was conducted in Italy, including 205 patients who had undergone neoadjuvant chemotherapy + trastuzumab. Pathological complete response (pCR) was obtained in 46.8% of these patients. In multivariate logistic regression models, non-luminal/HER2+ tumors and length of neoadjuvant trastuzumab treatment were independent predictors of pCR (*p* = 0.0001 and *p* = 0.03, respectively). Median event-free survival (EFS) and cancer-specific survival (CSS) were not reached at the time of the analysis. In multivariate analysis, non-luminal/HER2+ subgroup and pathological (p) stage II–III at surgery were the only variables significantly associated with worse long-term outcome (*p* = 0.01 and *p* = 0.01 for EFS and CSS by non-luminal/HER2+ subgroup and *p* = 0.0001 and *p* = 0.001 for EFS and CSS by p-stage at surgery, respectively) [9].

A study from the Southeast Netherlands Breast Cancer Consortium reported the real-life use and effectiveness of adjuvant trastuzumab in eBC patients, compared to standard chemotherapy without trastuzumab. Trastuzumab was delivered for a median of 17 cycles (range 2–37). Five-year DFS was 80.7%, while 5-year OS rates were 90.7% in patients treated with trastuzumab; both these outcomes were statistically improved with respect to those of the cohort not treated with trastuzumab [19].

Real-world data for eBC HER2+ patients were reported also from India, in a manuscript published by Adusumilli and colleagues. Out of a total of 885 patients, 212 resulted in HER2+ but only 76 (35.8%) received trastuzumab plus chemotherapy, due to financial issues. Patients having received trastuzumab with chemotherapy showed longer 5-year DFS and OS compared to those having received chemotherapy alone [20].

An additional real-world, multicenter study was conducted by our group to assess outcomes of HER2+ eBC patients in the pre-trastuzumab and post-trastuzumab eras (RETROHER study). Nine hundred and twenty-five consecutive HER2+ eBC patients treated with adjuvant chemotherapy were retrospectively recruited. Patients who had received adjuvant chemotherapy alone (cohort A, 352 patients), and patients who had received adjuvant chemotherapy followed by or combined with trastuzumab (cohort B, 573 patients) were analyzed. The median duration of trastuzumab treatment was 52 weeks (range, 1–104) The survival outcomes were significantly less favorable in the cohort A than in the cohort B (*p* = 0.0001). The benefit derived from the addition of trastuzumab was independent on nodal status and hormonal receptors expression. Interestingly, a subgroup analysis including 163 ‘‘triple positive’’ (TP) tumors with high levels of estrogen receptor (ER) and progesterone receptor (PgR), suggested that the addition of trastuzumab to adjuvant chemotherapy and hormonal therapy did not translate into better outcomes [21]. A subsequent analysis was performed by our group to better investigate the role of hormonal receptors (HRs) in HER2+ eBC [22]. We retrospectively identified 872 TP eBC patients treated with adjuvant chemotherapy alone or plus trastuzumab. Trastuzumab improved long-term outcomes in all the subsets analyzed, but the effect on breast cancer specific survival in tumors expressing both HRs in >30% of cells, and even on relapse free survival (RFS) in tumors with both HRs expressed in >50% of cells was not significant. Multivariate analysis of RFS confirmed a significant interaction between trastuzumab and ER expression, with benefit confined to patients whose tumors expressed ER in ≤50% of cells.

Also in the adjuvant setting, an interesting Italian experience was published by Mustacchi G. et al, focusing on relapse and discontinuation rates in a cohort of 1245 patients treated with adjuvant trastuzumab plus chemotherapy for HER2+ eBC. Nevertheless, the 21.3% of patients were excluded from adjuvant therapy because of comorbidities, age, toxicity or other reasons. In this subgroup, the relapse rate was higher than in chemotherapy treated patients (22.6% vs. 10.9%). However, among patients having received adjuvant trastuzumab plus chemotherapy, a correlation was seen between DFS and ER status [23].

The Promher Study was an observational experience on adjuvant therapy for HER2+, pT1a-b pN0 BC. The majority of patients (66%) underwent adjuvant chemotherapy plus trastuzumab. DFS was significantly higher in patients treated with adjuvant chemotherapy, with or without trastuzumab, than patients who did not receive systemic therapy [24].

A Japanese retrospective observational study investigated the survival rates and clinical features of 829 HER2+ eBC treated with neoadjuvant chemotherapy plus trastuzumab. The pathologic complete response rate was 51%, higher in the ER/PgR negative subgroup. Multivariate analysis revealed as independent predictors of low DFS the nodal status, nuclear grade and the pCR [25].

Finally, a significant contribution to this topic in the literature is given by a German retrospective study that enrolled about 4000 patients affected by HER2+ eBC treated with trastuzumab. Some of the included patient subgroups were not commonly presented in phase III trials (e.g., elderly patients and patients with stage I disease). The overall survival rates at 3 and 5 years seem to confirm the advantage conferred by trastuzumab use in eBC [26].

### 4.2. Metastatic Breast Cancer

In the metastatic setting, evidence from the real-world derive mostly from observational studies conducted in the “pre-pertuzumab” era, performed before the CLEOPATRA study [43]. This latter trial established the double HER2 blockade therapy with pertuzumab + trastuzumab and taxane-based chemotherapy as the standard first line treatment in patients with HER2+ MBC. In addition, since other HER2-targeted agents are now available in this setting (trastuzumab-emtansine, lapatinib), the use of trastuzumab as a single agent plus chemotherapy in first or subsequent lines is now restricted to selected patients.

The LHORA study has been previously mentioned. This was a multicenter observational trial including HER2+ MBC “long responders” patients treated with first line trastuzumab-therapy, with a PFS >3 years. The median duration of first-line trastuzumab was 4.5 years (range: 0.8–12.1 years), combined with paclitaxel or docetaxel. In these patients, median PFS was 6.4 years (95%CI:[5.7; Not Reached]) [13].

The real-world outcomes and treatment patterns in an Australian cohort (2001–2016) were the subject of three studies. First, an overall analysis performed in HER2+ MBC patients treated with trastuzumab was published: the clinical outcomes for trastuzumab in this heterogeneous real-world population were reassuringly comparable to those obtained from prospective clinical trials. Furthermore, authors stratified outcomes in two groups based on year of treatment initiation: 2001–2008 and 2009–2015. The duration of Trastuzumab treatment is longer in the second group than the first one [27]. Second, an analysis of long-term survival was performed: among 4177 women having initiated trastuzumab for HER2+ MBC, 1082 (26%) survived for at least (≥) 5 years. Median time on trastuzumab therapy was 58.9 months (range: 27.6–88.1 months) [28]. In addition, Parkinson and colleagues reported that patients from the real-world setting in whom trastuzumab was administered with a concomitant chemotherapy generally experienced longer OS than those treated with trastuzumab as a monotherapy [29].

An intriguing theme concerning the survival outcome of TP MBC patients (HER2+/hormone receptor positive) was analyzed by Tripathy and colleagues [30]. Patients treated with first line trastuzumab plus hormonal therapy had significantly longer PFS than patients who received hormonal therapy alone (13.8 vs. 4.8 months; [HR]: 0.37, 95% [CI]: 0.22–0.60); a non-significant reduction in OS was also observed. Compared to patients who received first-line trastuzumab plus chemotherapy, patients who received first-line trastuzumab plus chemotherapy and hormonal therapy had longer median PFS (20.4 months vs. 9.5 months; adjusted HR: 0.53, 95% CI: 0.42–0.68). Moreover, a statistically significant reduction in the risk of death was observed (HR: 0.50, 95% CI: 0.36–0.70). Sequential use of chemotherapy and hormonal therapy was associated with improved OS when compared with concurrent use (adjusted PFS HR: 0.81, 95% CI: 0.54–1.21; adjusted OS HR: 0.48, 95% CI: 0.26–0.89). These real-world data provide evidence that dual targeting of hormone receptors and HER2 receptors is associated with significantly prolonged PFS and OS. We have previously mentioned a further study from an Italian cohort of 681 HER2+ MBC patients treated with trastuzumab, which reported that OS was 81.8%, 64.0%, 50.2%, 41.1% and 37.2% at 1, 2, 3, 4 and 5 years, respectively [14]. A further interesting real-world study focused on elderly patients with HER2+ MBC reported improved outcomes in terms of PFS and OS in patients treated with trastuzumab with respect to patients who did not receive it [31].

### 4.3. Early Breast Cancer and Metastatic Breast Cancer

A large Chinese study explored the disparities of trastuzumab use due to financial reasons. Overall, the improvement in survival outcomes for trastuzumab users versus non-trastuzumab users was evident in both eBC (HR for DFS 0.51, 95% [CI]: 0.505–0.744) and in MBC (HR for PFS 0.51, 95% CI: 0.418–0.606) [32].

## 5. Discussion

The addition of trastuzumab to standard treatments in both the early and metastatic settings has dramatically changed the natural course of the HER2+ breast disease and translated into significantly improved clinical outcomes. Large scale data obtained from prospective interventional trials demonstrated a topping safety profile for trastuzumab, with the lonely exception of some cardiac adverse events. These latter events are most likely related to the interference of this monoclonal antibody with cardiomyocyte metabolism and with the remodeling of the micro-vascular endothelium which supports myocardial tissue. This form of toxicity seems to be most frequently associated with an asymptomatic decline in the LVEF rather than CHF.

The large-scale use of trastuzumab, which has been increasingly sustained by its advantageous therapeutic index, has fueled a growing interest towards long-term safety data from intervention trials and encouraged the follow up update of studies addressing safety as primary endpoint.

In relation to the early breast cancer setting, we mainly focused on five RCTs, i.e., the HERA trial, NSABP protocol B-31, NCCTG N9831 trial, BCIRG 006 and FinHer study, wherein trastuzumab was used in the adjuvant setting [6,7,8,37,38,39,40,41,42]. The incidence of any type and any grade of cardiac adverse events was relatively heterogeneous across these five trials and ranged from 0.4% to 7.3%. Cardiotoxicity was significantly more frequent in patients having received trastuzumab compared to those who have not, except for the FinHer trial. In this latter study, patients who did not undergo trastuzumab treatment experienced higher incidence of HF (1.7% vs. 0.9%). The time points at which the incidence of cardiac adverse events was recorded varied from a minimum of 5 years (FinHer trial) to a maximum of 11 years (HERA trial). Data were in key with the lack of significant changes of cardiac toxicity in the long-term. Inconsistencies of the trastuzumab-related cardiotoxicity incidence across these trials maybe at least partly explained by slightly different patients’ populations and use of trastuzumab according to different protocols. Importantly, in all these five trials most of the registered cardiac events had a rapid resolution following trastuzumab cessation. Overall, most of these trials confirm that trastuzumab-related toxicity more often occurs during treatment and its increased rate is due to the treatment itself. In these regards, the FinHer trial reported an inconsistent result. However, this trial included a limited number of patients. Some data suggest that severe cardiotoxicity is less tightly related to the length of trastuzumab treatment compared to mild toxicity (HERA trial). A further key issue emerged from these same studies is that the modality in which trastuzumab is administered seems to have a significant impact on the cardiotoxicity rates. More specifically, its concomitant use with chemotherapy, as well as after an anthracycline treatment, increases the cardiac adverse event rates. In this regard, a randomized prospective study conducted by Buzdar and co-authors investigated the concomitant use of trastuzumab and anthracyclines in the neoadjuvant setting [44]. Besides the exceptional outcomes in terms of pCR with the addition of trastuzumab to the anthracyclines, data on safety were comforting, since no CHF events were reported and the incidence of asymptomatic decline of LVFE was comparable to the non-trastuzumab-treated counterpart. It is noteworthy that exposure to trastuzumab in this trial was 24 weeks. A subsequent study by Dawood et al. in this same setting confirmed this outcome also in patients having received standard duration of trastuzumab [45]. A recent Italian phase II trial also explored the use of trastuzumab concomitantly to taxane and anthracycline treatment in the neoadjuvant setting, showing no increase in the rates of clinically overt HF and higher percentage of pCR than usually reported in trials assessing consecutive administration of these agents [46]. All these studies reported a reversible and rapidly recovering LVEF values in most of the cases.

Besides the data obtained from the RCTs, evidence on the safety of trastuzumab in eBC also derives from several observational studies. Although, of outmost importance in driving therapeutic decisions, evidence from randomized clinical trials may not necessarily apply to cancer patients from the real word setting, whose characteristics may not fully reflect those of patients enrolled in the registrative trials. In specific referral to the topic of interest, i.e., cardiotoxicity, the existence of co-morbidities directly and/or indirectly related to a higher risk of cardiac impairment may significantly affect the frequency and outcome of cardiac events in patients exposed to trastuzumab in the real-word setting.

Data from the OHERA study, Goldhar et al. and a Dutch experience, which overall included more than 7000 patients, reported different degrees of cardiac events in 2.5% to 7.8% of the patients [10,11,12]. In the OHERA study, the median time to symptomatic CHF was 5.7 months, while Goldhar et al. reported that trastuzumab related HF was higher in the first 1.5 years, but not thereafter. These data suggest that a routine intensive instrumental cardiac function monitoring may not be necessary following completion of the adjuvant treatment. Consistent efforts have been recently made to identify molecular and/or clinical predictors of cardiac adverse events related to trastuzumab treatment. In the OHERA trial, age greater than 65 years, pre-existing cardiac pathologic conditions, hypertension, or LVEF ≤55% at baseline were associated with higher rates of trastuzumab-related cardiotoxicity. 

Fewer data are available from the neoadjuvant setting. This is mainly due to the lack of endpoints of cardiac safety in the pre-planned analyses of the related studies. However, the reported cardiac adverse event rates were similar to those observed in the adjuvant trials, i.e., on average 1%–5% [47,48,49].

Evidence on long-term trastuzumab safety in the metastatic setting is relatively scarce. The LHORA study reported a 2.2% trastuzumab-related cardiotoxicity in patients whose PFS was longer than 3 years and who had undergone an uninterrupted trastuzumab-containing I line treatment. The ROP Study reported on the lack of severe cardiac events even in the case of treatment administration for ≥5 years [15]. Conversely, a retrospective study conducted by Yong Sun et al. showed a 13% incidence of ventricular dysfunction due to first line trastuzumab-containing therapy, which was not different in patients receiving trastuzumab for less than one year or more than one year [18]. These long-term follow up studies suggest that, in patients having received trastuzumab therapy, the instrumental monitoring of the heart function should be carefully considered in order to detect possible late events. This may be particularly relevant in the frame of a new clinical reality wherein novel targeted therapies have allowed significantly increases in the rate of long-term DFS, OS and definitive cure in patients treated for an eBC. In addition, the current availability of “omics”-related platforms may allow for the identification of biomarkers of cardiac toxicity. The use of these innovative, groundbreaking techniques, possibly combined with “old-fashion” monitoring techniques of cardiac function, may greatly increase our potentials in the investigation of trastuzumab-related toxicity and help us meet our patients’ needs at an individual level.

Evidence on trastuzumab efficacy from the real-world settings is of key importance in the management of patients who more often exhibit a worse performance status, relevant co-morbidities, older age at diagnosis, symptomatic disease or a combination of the aforementioned features. Real-world patients’ populations are generally less selected compared to their RCTs counterpart and the evidence from these latter trials may be somewhat limited in its generalizability to the real-life settings. 

Some studies focused on aspects related to the real-world treatment and effectiveness of trastuzumab in the eBC. A retrospective study conducted by our group in 205 patients who underwent trastuzumab treatment in the neoadjuvant setting, showed a 46.8% pCR rate, which was consistent with the results from prospective trials [9]. As expected, HER2 “enriched” subtype and time of exposure to trastuzumab were independent predictors of higher rate of pCR. The real-life study conducted by the Southeast Netherlands Breast Cancer consortium confirmed what demonstrated in prior prospective trials with regards to the absolute 5-year DFS and OS in patients receiving trastuzumab in this setting (respectively, 80.7% and 90.7%), but also substantiated the fact that these outcomes are significantly improved with respect to patients who did not receive trastuzumab [19].

In some areas, the use of trastuzumab is still limited by relevant financial issues. In the study from Adusumilli et al, only 35.8% of patients with an indication to trastuzumab could receive this targeted agent [20]. These patients showed significantly longer DFS and OS compared to their counterpart. In the RETROHER study, the focus was on outcomes of adjuvant treatment in the real-world early setting [21]. A clear difference emerged between HER2-like (ER-/HER2 +) and "triple-positive" (ER/PgR+/HER2+) BC. Overall survival was significantly longer in the patients treated in the post-trastuzumab era. This advantage was independent from the nodal status, even if outcomes were not improved in the TP subgroup. The benefit confined to patients whose tumors expressed ER in ≤50% of cells was also confirmed by the retrospective study that our group conducted [22]. An Australian cohort provided data for three different studies by Daniels et al. and Parkinson et al. [27,28,29]. These trials showed that clinical outcomes in terms of PFS and OS were somewhat more heterogeneous in the real-world population, but comparable to those from the RCTs. These authors also confirmed that the combination with chemotherapy improves the performance of trastuzumab in terms of clinical outcomes. The Tripathy trial showed that also in the real-world, treatment with first line trastuzumab + chemotherapy in HER2+ER+MBC has better outcomes with the addition of hormone therapy in terms of PFS and OS and that hormone therapy should be given sequentially with respect to chemotherapy [30].

A study by Kaufman addressed the real-world effectiveness of trastuzumab in elderly HER2+ MBC showing a conserved advantage in PFS and OS with its use also in this group of patients [31]. More generally, in the metastatic setting, outcome data from the real-world derived mostly from observational studies conducted before the establishment of the double HER2 blockade therapy with pertuzumab + trastuzumab and taxane as the standard first line treatment in patients with HER2+ MBC. New strategies and studies addressing the safety of trastuzumab in this new scenario are of utmost importance. A particularly interesting topic to a research agenda may be a possible increase in cardiotoxicity due to the dual blockage. Moreover, the role of additional HER2 targeted agents, such as trastuzumab-emtansine and lapatinib, should be addressed in the same terms by future trials, since they have become the standard of treatment in the subsequent lines.

Nevertheless, trastuzumab remains the cornerstone in HER2+ BC and is widely used as a single agent all over the world. Moreover, the development of several trastuzumab bio-similar drugs, which have recently entered the clinical practice, will hopefully downsize treatment costs.

## 6. Conclusions

Data of long-term safety of trastuzumab in HER+ BC confirm the good safety profile of this drug. Cardiotoxicity remains a concern, particularly in the course of treatment and in the presence of specific patient-related features. Few additional cardiac events are registered in the long-term course. However, careful cardiological monitoring tailored on the individual patient needs is recommended also in years following treatment with trastuzumab, particularly in high-risk subgroups. In addition, trastuzumab confirms its effectiveness in the real-world setting, both in eBC and MBC, based on data which are more easily generalizable to the clinical practice since deriving from non-selected populations. Further long-follow up data are needed to further reinforce the currently available evidence on the topics of interest.

## Figures and Tables

**Table 1:** Studies included for long-term safety.

	Study [Reference]	Median Follow-Up	Total Patients (N)	Arm and Patients (N)	Duration of H Administration	Cardiotox Secondary	Cardiotox Primary
eBC	HERA trial (BIG 1-01) [6]	11 years	5099	1 year H (1702)	1 year	4.4%	1%
				2 years H (1700)	2 years	7.3%	1%
				none (1697)	none	0.9%	0.1%
	NSABP B-31 [7]	7 years	1861	AC → P + H (960)	1 year	5.3%	3.9%
				AC → P (901)	none	2.0%	1.5%
	NCCTG N9831 (Alliance) [7]	6 years	1944	AC → P (664)	None	-	0.6%
				AC → P → H (710)	1 year	-	2.8%
				AC → P+H (570)	1 year	-	3.4%
	BCIRG 006 study [8]	65 months	3222	AC → T (1073)	None	11.2%	0.7%
				AC → T+H (1074)	1 year	18.6%	2.0%
				TCH (1075)	1 year	9.4%	0.4%
	FinHer Trial [9]	62 months	232	FEC → T or V (116)	None	10.5%	1.7%
				FEC → T or V + H (115)	9 weeks	6.8%	0.9%
	Final analysis of the OHERA study [10]	5 years	3733	H + cht	11.8 months	17.5%	2.8%
	The Temporal Risk of HF Associated with Adjuvant Trastuzumab in BC Patients: A Population Study [11]	5.9 years	19074	H + cht (3371)	1 year		5.2%
				Cht (15,703)	none		2.5%
	Cardiotoxicity and Cardiac Monitoring During Adjuvant Trastuzumab in Daily Dutch Practice: A Study of the Southeast Netherlands Breast Cancer Consortium [12]	5 years	230	H + cht	1 year	3.9%	8.7%
MBC	LHORA study (long responder, first line PFS >3 years) [13]	8 years	154	first line treatment with H + cht	4.5 years	2.2%	0.7%
	Trastuzumab for HER2+ metastatic breast cancer in clinical practice: Cardiotoxicity and overall survival [14]	5 years	681	Any treatment with H	-	7.2%	
	The Rollover Protocol (ROP) Study [15]	>5 years	19	Any treatment with H	8.7 years	0%	
	Long-term outcome of HER2 positive metastatic breast cancer patients treated with first-line trastuzumab [16]	10 years	215	first line treatment with H + cht	2.6 years	13%	2%
e/M BC	Long-term tolerance and cardiac function in breast cancer patients receiving trastuzumab therapy [17]	5 years	94	Adj or metastatic H	15.7 months	12.8%	-
	Evaluation of LVEF in Breast Cancer Patients Undergoing Long-Term Trastuzumab Treatment [18]	5 years	105	Adj or metastatic H	15.7 months	5.4%	

LEGEND: N = number. H = Herceptin, Trastuzumab. Cardiotox = cardiotoxicity. AC = anthracyclines + cyclophosphamide. P = paclitaxel, taxol. T = Taxotere, docetaxel. TCH: Taxotere + Carboplatin + Herceptin. FEC: 5-fluorouracyl + Epirubicin + Cyclophosphamide. V = vinorelbine. CHT = chemotherapy. eBC = early breast cancer. MBC = metastatic breast cancer.

**Table 2:** Studies included in real world effectiveness analysis.

	Study [Reference]	Median Follow-Up	Patients (N)	Arm And Patients (N)	Duration of H Administration	DFS/PFS/RFS (% or Median)	OS/BCSS (% or Median)
eBC	Effectiveness of neoadjuvant trastuzumab and cht in HER2+ BC [9]	32 months	205	NACT + H	24 weeks (median)	5 y DFS 73.3%	5 y CSS: 82.8%
	Real-Life Use and Effectiveness of Adjuvant Trastuzumab in eBC Patients: A Study of the Southeast Netherlands Breast Cancer Consortium [19]	5 years	476	Cht + H (230)	17 cycles (median)	5 y DFS 80.7%	5 y OS: 90.7%
				Cht (246)	-	5 y DFS 68.2%	5 y OS: 77.4%
	Treatment Challenges and Survival Analysis of Human Epidermal Growth Factor Receptor 2-positive Breast Cancer in Real World [20]	7 years	212	Cht + H (76)	NA	5 y DFS 92%	5 y OS: 90.5%
				Cht (136)	-	5 y DFS 52.6%	5 y OS: 41.7%
	The RETROHER study [21]	65 months	925	Cht + H (573)	52 weeks (median)	5 y DFS 88.6%	OS 5 y: 96%; 10 y: 93.3%
				Cht (352)	-	5 y DFS 71%	OS 5 y: 88.4%; 10 y: 72.3%
	“Triple positive” early breast cancer: an observational multicenter retrospective analysis of outcome [22]	78 months	872	Cht + H (506)	52 weeks (median)	5 y RFS 89.9%	BCSS 5 y: 97.4%; 10 y: 92%
				Cht (366)	-	5 y RFS 78.2%	BCSS 5 y: 93.9%; 10 y: 82%
	Observational study on adjuvant trastuzumab in HER2-positive early breast cancer patients [23]	50 months	1245	Cht + H	NA	10.9% RFS	-
				No adj therapy	-	22.6% RFS	-
	The Promher Study [24]	39 months	303	Cht + H (204)	NA	5y DFS 95%	-
				Cht alone (65)	-	5y DFS 94.3%	-
				No adj therapy (34)	-	5y DFS 69.6%	-
	JBCRG-C03 study [25]	42 months	829	NACT + H	NA	3y DFS 87%	-
	Trastuzumab in HER2+ eBC: Results of a Prospective, Noninterventional Study on Routine Treatment Between 2006 and 2012 in Germany [26]	39 months	4027	Cht + H	18 cycles (median)	3y DFS 90% 5y DFS 82.8%	3y OS 96.8% 5y OS 90%
MBC	LHORA study (long responder, first line PFS > 3 y) [13]	-	128	Cht + H	5.3 y (median)	Median PFS 6.4y	-
	Trastuzumab for metastatic breast cancer: Real world outcomes from an Australian whole-of-population cohort (2001–2016) [27]	6.7 years	5899	2001–08 (3122)	15 m (median)	-	Median 27 m
				2009–15 (2777)	18.4 m (median)	-	Median 37.7 m
	Real-World Evidence: A Comparison of the Australian Herceptin Program and Clinical Trials of Trastuzumab for HER2+ Metastatic Breast Cancer [28]	1.7 years	3617	H + cht	51.9 weeks (median)	Median PFS 8.3 m	Median 29.8 m
	Long-term survival in trastuzumab-treated patients with HER2- positive metastatic breast cancer: real-world outcomes and treatment patterns in a whole-of-population Australian cohort (2001–2016) [29]	9.4 years	1082	H + cht alive > 5 years from initial treatment	58.9 m (median)	-	Median 78.9 m (from 5th year)
	First-Line Treatment Patterns and Clinical Outcomes in Patients With HER2+ and Hormone Receptor-Positive MBC From registHER [30]	27 months	964	HR+/HER2+ (530)	NA	Median PFS 11.7 m	Median 41.5 m
				HR−/HER2+ (434)	NA	Median PFS 8.8 m	Median 28.6 m
	Trastuzumab for HER2+ metastatic breast cancer in clinical practice: Cardiotoxicity and overall survival [14]	5 years	681	H + cht	NA	-	5 y: 37.2%
	Treatment patterns and clinical outcomes in elderly patients with HER2-positive metastatic breast cancer from the registHER observational study [31]	27 months	209	H in first line	NA	Median PFS 11.7 m	Median 31.2 m
				No H in first line	-	Median PFS 4.6 m	Median 29.5m
e/MBC	Disparities of Trastuzumab Use in Resource-Limited or Resource-Abundant Regions and Its Survival Benefit on HER2 Positive Breast Cancer: A Real-World Study from China [32]	-	1139	eBC, yes H (397)	12 m	Median DFS 30 m	-
				eBC, no H (486)	-	Median DFS 21 m	-
				MBC, yes H (366)	10 m	Median PFS 13 m	-
				MBC, no H (354)	-	Median PFS 7 m	-

LEGEND: N = number. DFS = disease free survival. PFS = progression free survival. RFS = recurrence free survival. OS = overall survival. CSS = cancer specific survival. BCSS = breast cancer specific survival. NACT = neoadjuvant chemotherapy. H = Herceptin, Trastuzumab. Y = years. M = months. CHT = chemotherapy. HR+ = hormone receptor positive. HR− = hormone receptor negative. HER2+ = human epidermal growth factor receptor 2 positive. eBC = early breast cancer. MBC = metastatic breast cancer. NA = not available.

## References

[1] Owens M.A., Horten B.C., Da Silva M.M. (2004). HER2amplification ratios by fluorescence in situ hybridization and correlation with immunohistochemistry in a cohort of 6556 breast cancer tissues. Clin. Breast Cancer.

[2] Slamon D., Clark G., Wong S., Levin W., Ullrich A., McGuire W. (1987). Human breast cancer: correlation of relapse and survival with amplification of the HER-2/neu oncogene. Science.

[3] Viani G.A., Afonso S.L., Stefano E.J., De Fendi L.I., Soares F.V. (2007). Adjuvant trastuzumab in the treatment of HER2-positive early breast cancer: A meta-analysis of published randomized trials. BMC Cancer.

[4] Im Y., Odarchenko P., Grecea D., Komov D., Anatoliy C.V., Gupta S., Shparyk Y.V., Caguioa P.B., Makhson A., Krasnozhon D. (2013). Double-blind, randomized, parallel group, phase III study to demonstrate equivalent efficacy and comparable safety of CT-P6 and trastuzumab, both in combination with paclitaxel, in patients with metastatic breast cancer (mbc) as first-line treatment. J. Clin. Oncol..

[5] Stebbing J., Baranau Y.V., Baryash V., Manikhas A., Moiseyenko V., Dzagnidze G., Javrid E., Boliukh D., Stroyakovskiy D., Pikiel J. (2017). Double-blind, randomized phase III study to compare the efficacy and safety of CT-P6 trastuzumab biosimilar candidate versus trastuzumab as neoadjuvant treatment in her2 positive early breast cancer (ebc). J. Clin. Oncol..

[6] Cameron D., Piccart-Gebhart M.J., Gelber R.D., Procter M., Goldhirsch A., De Azambuja E., Castro G., Untch M., Smith I., Gianni L. (2017). 11 years’ follow-up of trastuzumab after adjuvant chemotherapy in HER2-positive early breast cancer: Final analysis of the HERceptin Adjuvant (HERA) trial. Lancet.

[7] Romond E.H., Perez E.A., Bryant J., Suman V.J., Geyer C.E., Davidson N.E., Tan-Chiu E., Martino S., Paik S., Kaufman P.A. (2005). Trastuzumab plus adjuvant chemotherapy for operable HER2-positive breast cancer. N. Engl. J. Med..

[8] Slamon D., Eiermann W., Robert N., Pienkowski T., Martín M., Press M., Mackey J., Glaspy J., Chan A., Pawlicki M. (2011). Adjuvant trastuzumab in HER2-positive breast cancer. N. Engl. J. Med..

[9] Natoli C., Vici P., Sperduti I., Grassadonia A., Bisagni G., Tinari N., Michelotti A., Zampa G., Gori S., Moscetti L. (2013). Effectiveness of neoadjuvant trastuzumab and chemotherapy in HER2-overexpressing breast cancer. J. Cancer Res. Clin. Oncol..

[10] Lidbrink E., Erfan J., Chmielowska E., Otremba B., Bouhlel A., Lauer S., Hermoso M.L., Nüesch E., Shing M., Misra V. (2017). OHERA: A real world study of cardiac events in >3700 patients with her2-positive early breast cancer treated with trastuzumab: Final analysis. Ann. Oncol..

[11] Goldhar H.A., Yan A.T., Ko D.T., Earle C.C., Tomlinson G.A., Trudeau M.E., Krahn M.D., Krzyzanowska M.K., Pal R.S., Brezden-Masley C. (2015). The Temporal Risk of Heart Failure Associated With Adjuvant Trastuzumab in Breast Cancer Patients: A Population Study. J. Natl. Cancer Inst..

[12] Seferina S.C., De Boer M., Derksen M.W., Berkmortel F.V.D., Van Kampen R.J., Van De Wouw A.J., Joore M., Peer P.G., Voogd A.C., Tjan-Heijnen V.C. (2016). Cardiotoxicity and Cardiac Monitoring During Adjuvant Trastuzumab in Daily Dutch Practice: A Study of the Southeast Netherlands Breast Cancer Consortium. Oncologist.

[13] Spano J.P., Beuzeboc P., Coeffic D., Arnould L., Lortholary A., Andre F., Ferrero J.M. (2015). Long term HER2+ metastatic breast cancer survivors treated by trastuzumab: Results from the French cohort study LHORA. Breast.

[14] Rossi M., Carioli G., Bonifazi M., Zambelli A., Franchi M., Moja L., Zambon A., Corrao G., La Vecchia C., Zocchetti C. (2016). Trastuzumab for HER2+ metastatic breast cancer in clinical practice: Cardiotoxicity and overall survival. Eur. J. Cancer.

[15] Müller V., Clemens M., Jassem J., Al-Sakaff N., Auclair P., Nüesch E., Holloway D., Shing M., Bang Y.-J. (2018). Long-term trastuzumab (Herceptin®) treatment in a continuation study of patients with HER2-positive breast cancer or HER2-positive gastric cancer. BMC Cancer.

[16] Yeo B., Kotsori K., Mohammed K., Walsh G., Smith I. (2015). Long-term outcome of HER2 positive metastatic breast cancer patients treated with first-line trastuzumab. Breast.

[17] Huang P., Dai S., Ye Z., Liu Y., Chen Z., Zheng Y., Shao X., Lei L., Wang X. (2017). Long-term tolerance and cardiac function in breast cancer patients receiving trastuzumab therapy. Oncotarget.

[18] Sun Y., Li T., Zhang Y., Zhang Q. (2016). Evaluation of Left Ventricular Ejection Fractions in Breast Cancer Patients Undergoing Long-Term Trastuzumab Treatment. Med. Sci. Monit..

[19] Seferina S.C., Lobbezoo D.J.A., De Boer M., Dercksen M.W., Berkmortel F.V.D., Van Kampen R.J.W., Van De Wouw A.J., De Vries B., Joore M.A., Peer P.G.M. (2015). Real-Life Use and Effectiveness of Adjuvant Trastuzumab in Early Breast Cancer Patients: A Study of the Southeast Netherlands Breast Cancer Consortium. Oncologist.

[20] Adusumilli P., Konatam M., Gundeti S., Bala S., Maddali L.S. (2017). Treatment Challenges and Survival Analysis of Human Epidermal Growth Factor Receptor 2-positive Breast Cancer in Real World. Indian J. Med. Paediatr. Oncol..

[21] Vici P., Pizzuti L., Natoli C., Moscetti L., Mentuccia L., Vaccaro A., Sergi D., Di Lauro L., Trenta P., Seminara P. (2014). Outcomes of HER2-positive early breast cancer patients in the pre-trastuzumab and trastuzumab eras: A real-world multicenter observational analysis. The RETROHER study. Breast Cancer Res. Treat..

[22] Vici P., Pizzuti L., Sperduti I., Frassoldati A., Natoli C., Gamucci T., Tomao S., Michelotti A., Moscetti L., Gori S. (2016). Triple positive" early breast cancer: An observational multicenter retrospective analysis of outcome. Oncotarget.

[23] Mustacchi G., Puglisi F., Molino A., Crivellari D., Ghiotto C., Ferro A., Brunello A., Saracchini S., Turazza M., Cretella E. (2015). Observational study on adjuvant trastuzumab in HER2-positive early breast cancer patients. Future Oncol..

[24] Gori S., Inno A., Fiorio E., Foglietta J., Ferro A., Gulisano M., Pinotti G., Gubiotti M., Cavazzini M.G., Turazza M. (2015). The Promher Study: An Observational Italian Study on Adjuvant Therapy for HER2-Positive, pT1a-b pN0 Breast Cancer. PLoS ONE.

[25] Takada M., Ishiguro H., Nagai S., Ohtani S., Kawabata H., Yanagita Y., Hozumi Y., Shimizu C., Takao S., Sato N. (2014). Survival of HER2-positive primary breast cancer patients treated by neoadjuvant chemotherapy plus trastuzumab: A multicenter retrospective observational study (JBCRG-C03 study). Breast Cancer Res. Treat..

[26] Dall P., Koch T., Göhler T., Selbach J., Ammon A., Eggert J., Gazawi N., Rezek D., Wischnik A., Hielscher C. (2017). Trastuzumab in Human Epidermal Growth Factor Receptor 2-Positive Early Breast Cancer: Results of a Prospective, Noninterventional Study on Routine Treatment Between 2006 and 2012 in Germany. Oncologist.

[27] Daniels B., Kiely B.E., Lord S.J., Houssami N., Lu C.Y., Ward R.L., Pearson S.-A. (2018). Trastuzumab for metastatic breast cancer: Real world outcomes from an Australian whole-of-population cohort (2001-2016). Breast.

[28] Daniels B., Kiely B.E., Lord S.J., Houssami N., Lu C.Y., Ward R.L., Pearson S.-A. (2018). Long-term survival in trastuzumab-treated patients with HER2-positive metastatic breast cancer: Real-world outcomes and treatment patterns in a whole-of-population Australian cohort (2001–2016). Breast Cancer Res. Treat..

[29] Parkinson B., Viney R., Haas M., Goodall S., Srasuebkul P., Pearson S.-A. (2016). Real-World Evidence: A Comparison of the Australian Herceptin Program and Clinical Trials of Trastuzumab for HER2-Positive Metastatic Breast Cancer. PharmacoEconomics.

[30] Tripathy D., Kaufman P.A., Brufsky A.M., Mayer M., Yood M.U., Yoo B., Quah C., Yardley D., Rugo H.S. (2013). First-line treatment patterns and clinical outcomes in patients with HER2-positive and hormone receptor-positive metastatic breast cancer from registHER. Oncologist.

[31] Kaufman P.A., Wang L.I., Quah C.S., Brufsky A.M., Mayer M., Rugo H.S., Tripathy D., Yood M.U., Feng S., Yardley D.A. (2012). Treatment patterns and clinical outcomes in elderly patients with HER2-positive metastatic breast cancer from the registHER observational study. Breast Cancer Res. Treat..

[32] Li J., Wang S., Wang Y. (2017). Disparities of Trastuzumab Use in Resource-Limited or Resource-Abundant Regions and Its Survival Benefit on HER2 Positive Breast Cancer: A Real-World Study from China. Oncologist.

[33] Russell S.D., Blackwell K.L., Lawrence J., Pippen J.E., Roe M.T., Wood F., Paton V., Holmgren E., Mahaffey K.W. (2010). Independent adjudication of symptomatic heart failure with the use of doxorubicin and cyclophosphamide followed by trastuzumab adjuvant therapy: A combined review of cardiac data from the National Surgical Adjuvant breast and Bowel Project B-31 and the North Central Cancer Treatment Group N9831 clinical trials. J. Clin. Oncol..

[34] Rochette L., Guenancia C., Gudjoncik A., Hachet O., Zeller M., Cottin Y., Vergely C. (2015). Anthracyclines/trastuzumab: New aspects of cardiotoxicity and molecular mechanisms. Trends Pharmacol. Sci..

[35] Chien J., Rugo H.S. (2010). The cardiac safety of trastuzumab in the treatment of breast cancer. Expert Opin. Drug Saf..

[36] Zeglinski M., Ludke A., Jassal D.S., Singal P.K. (2011). Trastuzumab-induced cardiac dysfunction: A “dual-hit”. Exp. Clin. Cardiol..

[37] Piccart-Gebhart M.J., Procter M., Leyland-Jones B. (2005). Trastuzumab after adjuvant chemotherapy in HER2-positive breast cancer. N. Engl. J. Med..

[38] De Azambuja E., Procter M.J., Van Veldhuisen D.J., Agbor-Tarh D., Metzger-Filho O., Steinseifer J., Untch M., Smith I.E., Gianni L., Baselga J. (2014). Trastuzumab-associated cardiac events at 8 years of median follow-up in the Herceptin Adjuvant trial (BIG 1-01). J. Clin. Oncol..

[39] Romond E.H., Jeong J.-H., Rastogi P., Swain S.M., Geyer C.E., Ewer M.S., Rathi V., Fehrenbacher L., Brufsky A., Azar C.A. (2012). Seven-year follow-up assessment of cardiac function in NSABP B-31, a randomized trial comparing doxorubicin and cyclophosphamide followed by paclitaxel (ACP) with ACP plus trastuzumab as adjuvant therapy for patients with node-positive, human epidermal growth factor receptor 2-positive breast cancer. J. Clin. Oncol..

[40] Ganz P.A., Romond E.H., Cecchini R.S., Rastogi P., Geyer C.E., Swain S.M., Jeong J.-H., Fehrenbacher L., Gross H.M., Brufsky A.M. (2017). Long-Term Follow-Up of Cardiac Function and Quality of Life for Patients in NSABP Protocol B-31/NRG Oncology: A Randomized Trial Comparing the Safety and Efficacy of Doxorubicin and Cyclophosphamide (AC) Followed by Paclitaxel With AC Followed by Paclitaxel and Trastuzumab in Patients With Node-Positive Breast Cancer With Tumors Overexpressing Human Epidermal Growth Factor Receptor 2. J. Clin. Oncol..

[41] Advani P.P., Ballman K.V., Dockter T.J., Colon-Otero G., Perez E.A. (2016). Long-Term Cardiac Safety Analysis of NCCTG N9831 (Alliance) Adjuvant Trastuzumab Trial. J. Clin. Oncol..

[42] Joensuu H., Bono P., Kataja V., Alanko T., Kokko R., Asola R., Utriainen T., Turpeenniemi-Hujanen T., Jyrkkiö S., Moykkynen K. (2009). Fluorouracil, epirubicin, and cyclophosphamide with either docetaxel or vinorelbine, with or without trastuzumab, as adjuvant treatments of breast cancer: Final results of the FinHer Trial. J. Clin. Oncol..

[43] Swain S.M., Baselga J., Kim S.-B., Ro J., Semiglazov V., Campone M., Ciruelos E., Ferrero J.-M., Schneeweiss A., Heeson S. (2015). Pertuzumab, trastuzumab, and docetaxel in HER2-positive metastatic breast cancer. N. Engl. J. Med..

[44] Buzdar A.U., Ibrahim N.K., Francis D., Booser D.J., Thomas E.S., Theriault R.L., Pusztai L., Green M.C., Arun B.K., Giordano S.H. (2005). Significantly higher pathologic complete remission rate after neoadjuvant therapy with trastuzumab, paclitaxel, and epirubicin chemotherapy: Results of a randomized trial in human epidermal growth factor receptor 2-positive operable breast cancer. J. Clin. Oncol..

[45] Dawood S., Gonzalez-Angulo A.M., Peintinger F., Broglio K., Symmans W.F., Kau S.W., Islam R., Hortobagyi G.N., Buzdar A.U. (2007). Efficacy and safety of neoadjuvant trastuzumab combined with paclitaxel and epirubicin: A retrospective review of the M. D. Anderson experience. Cancer.

[46] Pizzuti L., Barba M., Giannarelli D., Sergi D., Botti C., Marchetti P., Anzà M., Maugeri-Saccà M., Natoli C., Di Filippo S. (2016). Neoadjuvant Sequential Docetaxel Followed by High-Dose Epirubicin in Combination With Cyclophosphamide Administered Concurrently With Trastuzumab. The DECT Trial. J. Cell. Physiol..

[47] Untch M., Fasching P.A., Konecny G.E., Hasmüller S., Lebeau A., Kreienberg R., Camara O., Müller V., Du Bois A., Kühn T. (2011). Pathologic complete response after neoadjuvant chemotherapy plus trastuzumab predicts favorable survival in human epidermal growth factor receptor 2-overexpressing breast cancer: Results from the TECHNO trial of the AGO and GBG study groups. J. Clin. Oncol..

[48] Gianni L., Eiermann W., Semiglazov V., Manikhas A., Lluch A., Tjulandin S., Zambetti M., Vazquez F., Byakhow M., Lichinitser M. (2010). Neoadjuvant chemotherapy with trastuzumab followed by adjuvant trastuzumab versus neoadjuvant chemotherapy alone, in patients with HER2-positive locally advanced breast cancer (the NOAH trial): A randomised controlled superiority trial with a parallel HER2-negative cohort. Lancet.

[49] Untch M., Rezai M., Loibl S., Fasching P.A., Huober J., Tesch H., Bauerfeind I., Hilfrich J., Eidtmann H., Gerber B. (2010). Neoadjuvant treatment with trastuzumab in HER2-positive breast cancer: Results from the GeparQuattro study. J. Clin. Oncol..

